# Calcific Myonecrosis of the Leg: A Rare Entity

**DOI:** 10.3390/medicina55090542

**Published:** 2019-08-28

**Authors:** Andrea Angelini, Andreas F. Mavrogenis, Elisa Pagliarini, Giulia Trovarelli, Giuseppe Nicolò Fanelli, Rocco Cappellesso, Pietro Ruggieri

**Affiliations:** 1 Department of Orthopaedics and Orthopaedic Oncology, University of Padova, 35128 Padova, Italy; 2 First Department of Orthopaedics, National and Kapodistrian University of Athens, School of Medicine, 115 27 Athens, Greece; 3 Department of Medicine, Surgical Pathology & Cytopathology Unit, University of Padova, 35128 Padova, Italy

**Keywords:** calcific, myonecrosis, trauma

## Abstract

Calcific myonecrosis is a rare disease that has been shown to be a late sequela of trauma. This article presents a 68-year-old man with calcific myonecrosis of the leg 40 years after a tibial fracture complicated with peroneal nerve palsy. The soft tissue mass increased in size after another injury to the leg that occurred two years before his presentation. Physical examination at presentation showed a palpable extra-osseous mass at the anterior aspect of the left leg; the mass was not adherent to adjacent soft-tissues and bone, and it was painless but tender to palpation. Radiographs of the left leg showed extensive calcification at the soft-tissue of the anterior and posterior leg. An ultrasonography-guided trocar biopsy was done; histological findings were indicative of calcific myonecrosis. Given the benign entity of the lesion and known high rate of complications, he was recommended for no further treatment except for clinical and imaging observation. Located at the site of the biopsy, he experienced infection with drainage that eventually healed after six months with antibiotics and wound dressing changes. During the last follow-up examination, two years after diagnosis, the patient was asymptomatic without progression of the mass.

## 1. Introduction

Calcific myonecrosis is a rare disease that has been shown to be a late sequela of trauma [1,2]. The time of the onset of symptoms is significantly different from the initial injury and progresses slowly over a period of several years, making an accurate diagnosis difficult. First described more than 40 years ago by Gallie and Thomson [1], it is characterized by the replacement of muscle in one or more compartments with a fusiform mass or masses showing peripheral calcification and central liquefaction. It has been reported to develop primarily in the leg and has been attributed to trauma, such as a fracture accompanying ischemic symptoms and/or peripheral nerve injury [1,2,3,4,5,6,7,8,9,10,11,12,13,14,15,16,17,18,19,20,21,22,23,24,25,26,27]. Although not well understood, it is postulated that these lesions most likely result from post-traumatic ischemia and cystic degeneration of the muscle [19]. The plaque-like amorphous calcification pattern seen on radiographs is characteristic but not pathognomonic; the calcifications are usually linear in orientation and sheet-like, and present within the entire muscle or compartment, with mixed areas of radiolucency [19]. Smooth bony erosions may be present with minimal periosteal reaction. Occasionally, the erosions may be extensive and worrisome for a soft-tissue tumor; importantly, differential diagnosis should include a sarcoma due to its large size, growth potential and imaging characteristics [4,5,6,7,19].

To enhance the literature, this article presents a 68-year-old man with calcific myonecrosis of the leg 40 years after a tibial fracture. The soft tissue mass increased in size after another injury to the leg that occurred two years before his presentation. The diagnostic approach, differential diagnosis and treatment are discussed. Written informed consent for publishing this study was obtained from the patient.

## 2. Case Report

A 68-year-old man presented with a progressive painless swelling of his left leg. Past medical history revealed an ipsilateral proximal tibia and fibula fracture 40 years before; the tibial fracture was treated then with an open reduction and internal fixation with a plate and screws. Since then, he experienced a postoperative deficit of the left common peroneal nerve. Two years before, after a minor injury (a muscle strain) to the same leg, he noticed a palpable, painless mass that had increased in size substantially over the last five months.

**Figure 1:**
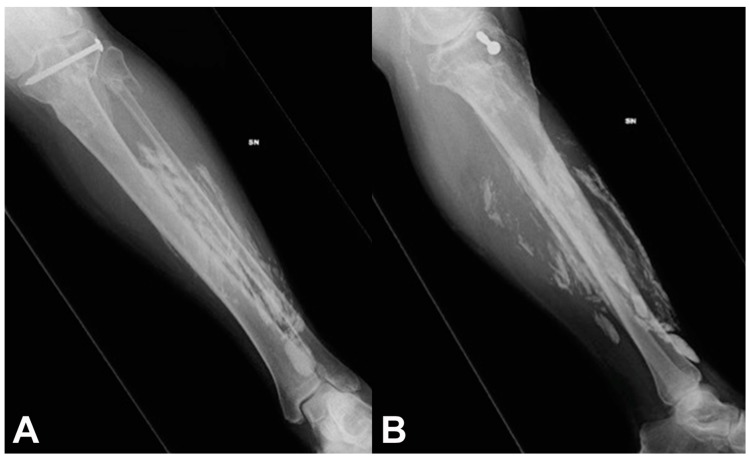
(**A**) Anteroposterior and (**B**) lateral radiographs of the left leg of a 68-year-old man with biopsy diagnosed calcific myonecrosis of the leg show extensive calcifications at the soft-tissue of the anterior and posterior leg.

Physical examination at presentation showed a palpable extra-osseous mass at the anterior aspect of the left leg; the mass was not adherent to adjacent soft-tissues and bone, and it was tender but painless to palpation. The skin was dry and not erythematous. Dorsiflexion of the left foot was weak secondary to the known left common peroneal nerve deficit. Radiographs of the left leg showed extensive calcification at the soft-tissue of the anterior and posterior leg (Figure 1).

**Figure 2:**
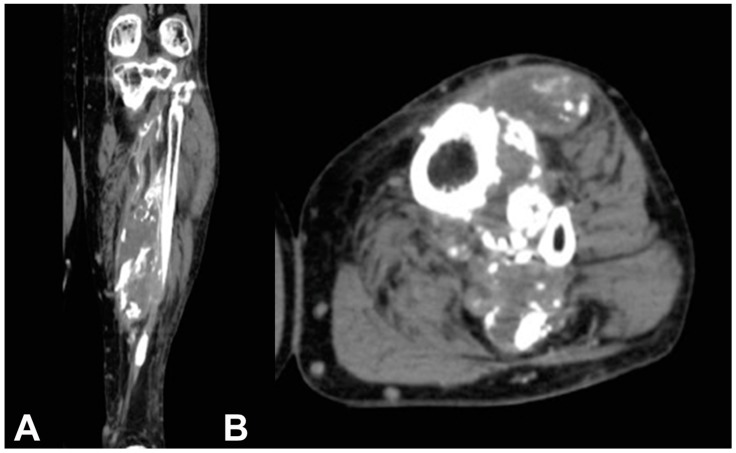
(**A**) Coronal and (**B**) axial CT scan of the leg show a calcified fusiform soft-tissue mass with peripheral plaque-like calcification linearly oriented through the muscles of the anterior and posterior leg compartment and interosseous membrane and marked erosion of the fibular cortex. The scalloping of the tibia suggests a slow growing process; central low signal density, and peripheral high density suggests fluid and peripheral calcification.

Computed tomography (CT) scan of the left leg showed a calcified fusiform soft-tissue mass with peripheral plaque-like calcification linearly oriented through the muscles of the anterior and posterior leg and extending through the interosseous membrane (Figure 2).

**Figure 3:**
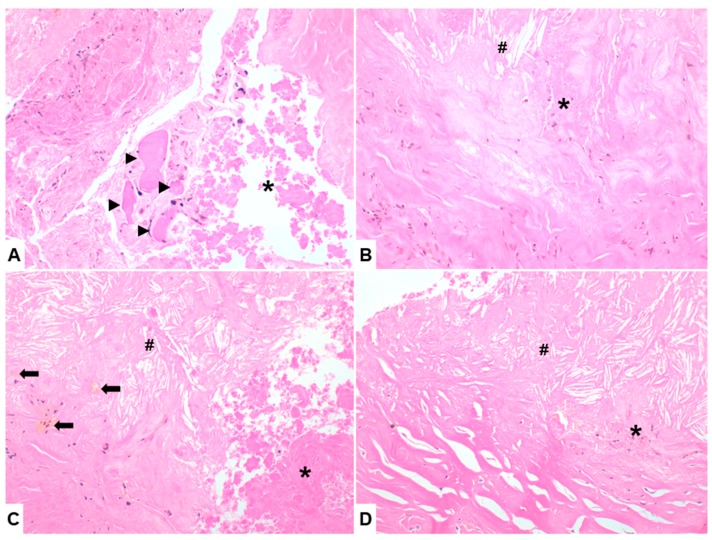
(**A**) Calcific myonecrosis is histologically characterized by degenerating myofibers that exhibit cell swelling, hyper-eosinophilia, loss of cross striation and fragmentation (arrowhead) with pyknotic nuclei. (**B**–**D**) Long standing degenerated regions variably can be accompanied by additional myopathic changes, such as extensive necrosis (asterisk) with prominent ghost skeletal muscle fibers, atrophy, sclero-hyalinized foci (top right (**A**,**B**), bottom left (**C**,**D**)) and inflammatory cells infiltration. Along the areas of more recent haemorrhage, hemosiderin deposits are present (arrow). Cholesterol crystals (diesis) with extensive dystrophic calcification are other distinguishing features.

Laboratory studies were within normal values. To exclude malignancy, an ultrasonography-guided trocar biopsy was done. Histological sections of the biopsy specimens showed multiple fragments of pultaceous s and partially calcified tissue, presence of sclero-hyaline and necrotic tissue with calcifications without any vital cells; histological findings were indicative of the diagnosis of calcific myonecrosis (Figure 3).

The patient was informed of the benign entity of the lesion. Given the known high rate of complications, he was recommended for no further treatment except for clinical and imaging observation. Located at the site of the biopsy, he experienced infection with drainage that eventually healed after six months with antibiotics and wound dressing changes. During the last follow-up examination, two years after diagnosis, the patient experiences no noticeable increase in the size of the mass, and diminished pain. Repeat radiographs of the leg were similar to those at presentation. He was advised to continue full activity and to monitor his leg for new symptoms or mass enlargement.

## 3. Discussion

Calcific myonecrosis is rarely reported in literature [1,2,3,4,5,6,7,8,9,10,11,12,13,14,15,16,17,18,19,20,21,22,23,24,25,26,27]; our literature search came out with 40 cases reported from the original description of the entity (Table 1). Age range varied significantly (range, 17–87 years); patients in their sixth decade of life were affected most frequently. There was a slight male predominance (33 male and 7 female patients), as in the present case. The most common area of calcific myonecrosis was the anterior compartment of the leg followed by the lateral and deep posterior compartments; a total of 37 cases were reported in the leg [1,2,4,5,6,8,9,10,11,12,13,14,15,16,17,18,19,20,21,23,24,25,26,27], two cases in the forearm [3,22], and one case in the foot [7].

A closed fracture or trauma often associated to compartment syndrome are the most common causes of calcific myonecrosis [1,2,3,5,6,7,8,9,10,11,12,13,14,15,16,17,19,20,21,22,23,24,25,26,27]; in two papers describing 14 cases, data on previous trauma were not available [4,18]. Trauma may consist of a fracture (26 cases), strain or ligamentous injury (10 cases), snake bite (2 cases) [16,23], and gunshot lesion (2 cases) [25,27]. A peroneal nerve injury was reported in nine cases [5,6,14,17,24,25], and a sciatic nerve injury in two cases [10,18]. Clinical presentation usually includes a painful soft tissue mass; occasionally, as in the present patient, the mass can be painless, or present with signs of infection [7,17]. The mean interval from trauma to occurrence of clinical symptoms is 37.6 years (range, 5 years to 59 years); the mean duration of clinical symptoms is 3 months (range, 10 days to 20 years) [17,24]. The patient presented herein had a history of tibia and fibula fracture 40 years before; however, he experienced the growing palpable mass only after the second injury to the leg, as previously reported in one case [26].

Radiographs and CT scans usually show fusiform soft-tissue masses with longitudinal peripheral plaque-like calcifications resembling an eggshell, and multiple fragmented calcifications involving the entire compartment [9,19]. CT scans confirm the compartmental involvement and more clearly illustrate the peripheral calcification and periosteal erosions [9,19]. Bone erosion could be observed with smooth periosteal reaction [3]. Magnetic resonance (MR) imaging usually shows an iso- or hyperintense soft-tissue lesion with central liquefaction and hypointense coarse calcifications without enhancement after gadolinium administration [6]. Peripheral ring enhancement on postcontrast fat-suppression T1-weighted MR imaging has also been observed [14].

The differential diagnosis should include soft-tissue sarcomas with a propensity for calcifications (mineralization) or ossifications (dense bone formation) such as synovial sarcoma, epithelioid sarcoma and extraskeletal osteosarcoma, as well as benign lesions such as myositis ossificans and inflammatory diseases [7,17,19]. Synovial sarcoma, epithelioid sarcoma and extraskeletal osteosarcoma may radiographically resemble calcific myonecrosis; however, the calcific pattern in myonecrosis has a peripheral distribution with a central core of necrosis that is different from sarcomas in which calcifications/ossifications are usually distributed throughout the mass [17]. Myositis ossificans usually show calcifications with the absence of an intralesional trabecular pattern and marrow signal on MR imaging or progressive enlargement of the mass [7]. Other benign entities resembling calcific myonecrosis are post-traumatic pseudoaneurysms, dermatomyositis, polymyositis, diabetic myonecrosis and tumoral calcinosis; in these cases, respective systemic symptoms are quite evident [2,10,26,28]. Biopsy should be used in most cases that clinical and imaging appearance resemble a sarcoma for histological diagnosis. However, the surgeon (and patient) should be aware that biopsy potentially leads to more problems than it is worth. Regarding either a closed or an open biopsy, complications may occur, most commonly infection and wound healing problems [7]. Therefore, asymptomatic patients with a stable lesion, typical medical history (remote fracture, compartment syndrome) and imaging, should be watched rather than biopsied.

When calcific myonecrosis is confirmed, treatment is controversial. Various treatments have been reported; some authors have recommended surgical excision for symptomatic patients [9,10], while others recommended aggressive debridement with flap coverage [6,7,15], even if several have ultimately been unsuccessful or associated with complications [4,6,7,18,23,24,26]. Infection is the most common complication, accounting for approximately 62% of the reported cases after a surgical operation or even a biopsy procedure [2,3,4,6,7,18,23,24,26]. When it occurs, infection should be treated with wide spectrum antibiotics for 6–8 weeks with a minimum of two weeks of intravenous administration [2,7,15]. Due to the high risk of infection, all authors agree that surgical intervention in asymptomatic patients should be avoided; conservative treatment with follow-up observation is the best option after documentation of the diagnosis. Malignant degeneration has not been reported.

## 4. Conclusions

To conclude, calcific myonecrosis is a benign entity that usually affects the leg secondary to trauma. Biopsy is not necessary in asymptomatic patients with a stable lesion, typical medical history (remote fracture, compartment syndrome) and imaging. Conservative treatment with clinical and radiographic follow-up is the best treatment approach due to the high risk for infection.

## Figures and Tables

**Table 1:** Summary of the published reports on calcific myonecrosis.

Study	Patients/Age, gender	Site	Symptoms (time)	Previous trauma (time before)	Nerve deficit	Biopsy	Complications	Infection	Treatment
Janzen et al. [6]	77M	Leg	Mass	Fibular fracture (52 years)	Peroneal	Incisional	Yes	Chronic drainage	n.a.
Zohman et al. [10]	49M	Leg	Mass	Ligamentous knee injury (30 years)	Sciatic	Incisional	No	–	Excision
Tuncay et al. [25]	64M	Leg	Mass (3 years)	Shotgun injury (42 years)	Peroneal	Incisional	No	–	Excision
Jassal et al. [26]	66M	Leg	Mass, pain	Ankle fracture (47 years)	–	Incisional	Yes	Cellulitis and drainage	Debridement + flap
Holobinko et al. [7]	57M	Leg	Mass, pain (2 years)	Tibia fracture (40 years)	Weakness	Incisional	Yes	Drainage	Debridement + flap
	67M	Leg	Mass (3 months)	Tibia fracture (51 years)	–	Incisional	No	Drainage	Debridement
	37M	Foot	Plantar drainage	Tibia fracture (21 years)	Hyperesthesia	Incisional	No	Drainage	Debridement + flap
Dhillon et al. [4]	7M, 3F; mean age 68 years (range, 40–82 years)	Leg	n.a.	Significant trauma to the leg (mean, 46 years; range 28–59 years)	n.a.	Needle (4 patients)	8 patients (prolonged recovery)	n.a.	Debridement (3 patients)
Larson et al. [3]	17M	Forearm	Mass, pain (4 months)	Crush injury (55 years)	–	Incisional	No	–	Excision
Ozbarlas et al. [12]	77M	Leg	Mass, pain (2 months)	Blunt trauma (5 years)	–	n.a.	n.a.	–	n.a.
Muramatsu et al. [5]	51M	Leg	Mass, pain (3 months)	Compartment syndrome. (35 years)	Peroneal	Needle	–	–	Debridement
	52M	Leg	Growing mass (1 month)	Tibia fracture (24 years)	Tibialis	Incisional	–	Osteomyelitis	Aspiration
	66M	Leg	Mass, pain (3 months)	Crush injury (40 years)	–	–	–	Yes	Aspiration
Okada et al. [14]	62M	Leg	Mass (5 months)	Squeezed trauma (43 years)	Peroneal	Incisional	–	–	Excision
Papanikolaou et al. [17]	54M	Leg	Mass (10 days)	Crush injury (7 years)	–	–	–	Yes	Debridement
	66M	Leg	Mass, pain (1 month)	Artery lesion (52 years)	Peroneal	–	–	Drainage, fever	Debridement, VACT
	84F	Leg	Infection	Crush injury (53 years)	–	–	–	Yes, fever	Debridement
Portabella et al. [18]	55M	Leg	n.a.	n.a.	Sciatic	Yes	–	–	–
	64M	Leg	n.a.	n.a.	Peroneal	–	Yes	Chronic drainage	Debridement, VACT
	54M	Leg	n.a.	n.a.	Peroneal	–	Yes	Chronic drainage	Debridement, VACT
	77M	Leg	n.a.	n.a.	–	–	–	Yes	Debridement
De Carvalho et al. [13]	69F	Leg	Mass (2 months)	Motor vehicle trauma	–	–	–	–	n.a.
	73M	Leg	Growing mass	Tibia fracture (57 years)	–	–	–	–	n.a.
Chun et al. [16]	53M	Leg	Growing mass	Snake bite (44 years)	–	–	–	–	n.a.
Jalil et al. [20]	43M	Leg	Mass, pain (1 month)	Tibia fracture (20 years)	–	–	–	–	Debridement
Karkhanis et al. [21]	60M	Leg	Growing mass (4 months)	–	–	–	–	–	–
Rynders et al. [22]	66M	Forearm	Growing mass (2 months)	Elbow fracture (57 years)	–	–	–	n.a.	n.a.
Yuenyongviwat et al. [23]	66F	Leg	Mass (10 years)	Snake bite (14 years)	Peroneal	Yes	Yes	Yes	Excision
Ukon et al. [24]	69F	Leg	Growing mass (20 years)	Fibular fracture (20 years)	–	Incisional	Yes	Chronic drainage	–
	76M	Leg	Growing mass (2 months)	Tibia, fib fracture (55 years)	Peroneal	Incisional	Yes	Yes	Debridement
Güven et al. [27]	66M	Leg	Mass, pain (12 months)	Compartment syndrome after gunshot injury of the thigh (35 years)	–	Excisional	–	–	–

M = male; F = female; n.a. = not available; VACT = Vacuum Assisted Closure Therapy.
